# Human Papillomavirus and Its Association With Cervical Cancer: A Review

**DOI:** 10.7759/cureus.57432

**Published:** 2024-04-01

**Authors:** Eman A Alrefai, Rawan T Alhejaili, Sohailah A Haddad

**Affiliations:** 1 Obstetrics and Gynaecology, Taibah University, Medina, SAU; 2 Medicine, Taibah University, Medina, SAU; 3 Medicine, Taibah University, Madina, SAU

**Keywords:** vaccination, pap smear, cervical cancer, infection, human papillomavirus

## Abstract

The human papillomavirus (HPV) belongs to the Papillomavirus family and is considered a non-enveloped virus. HPV affects individuals by causing both benign and malignant lesions. We aim to define HPV and its important characteristics, explain the relation between HPVs and cervical cancer, review its prevalence among Saudi women and their awareness of screening and prevention of cervical cancer, and focus on the importance of HPV vaccination. The persistence of HPV infection is the most important risk factor for the development of cervical cancer. HPVs cannot be cultured, and the identification of the virus is dependent on a variety of techniques, including immunology, serology, and molecular biology. Cervical cancer is the fourth most prevalent form of cancer in women worldwide, while it is considered the 12th type of cancer that affects Saudi women. Unfortunately, many studies have shown a lack of awareness regarding HPV infection, screening, and vaccination among the Saudi population in general, as well as among Saudi healthcare professionals. The HPV vaccine has a potent role in preventing people from getting infected with the virus, despite some previous clinical trials assessing the outcomes of therapeutic HPV vaccinations showing unsatisfactory results. While there is no doubt about the benefits of vaccines and their role in reducing the incidence of HPV infectious diseases, there are discrepancies in the evaluation of the safety of the HPV vaccine. In conclusion, HPV is an essential etiology of cervical cancer, and the expansion of public awareness about protective methods and threat factors associated with HPV infection is highly important.

## Introduction and background

Cervical cancer is the third most common malignancy in women worldwide. In Saudi Arabia, cervical cancer is the 12th most common cancer among women. Also, it is considered one of the most common cancers related to death [1]. Most cervical cancer cases are caused by the human papillomavirus (HPV), and it may be difficult to detect the infection early without screening [2].

HPV is a group of viruses that are extremely common worldwide. HPV is highly species-specific and does not infect other species. The most common transmission of HPV is sexual transmission; it could also be transmitted non-sexually and occasionally even through feces. In addition, early sexual activity, multiple sexual partners, and use of oral contraceptives, as well as low socioeconomic status and smoking, contribute to increasing the risk of acquiring the infection [3,4].

HPV infection can lead to cervical cancer, especially in certain epidermal sites, such as the skin or genital mucosa. Among all human cancers, 15% are caused by viral infections. The HPV accounts for approximately 600,000 cases of cancer of the cervix. There are more than 100 types of HPV and about 14 of them are associated with cancer [3]. Two HPV types (16 and 18) are considered high-risk HPVs that cause 70% of cervical cancers and precancerous cervical lesions [2].

## Review

Human papillomavirus

More than 120 HPV types have been identified. Among them, around 80 are considered non-mucosal/cutaneous genitalia viruses that appear as skin warts (in hands and feet). However, nearly 40 types are mucosal/genital, which are further subdivided into high-risk types, such as 16 and 18, and low-risk types, such as 6 and 11 [1-5].

Structure and morphology of HPV

Belonging to the Papillomavirus family, HPV is a non-enveloped virus with a compact circular double-stranded DNA structure measuring around 55 nm in size. Furthermore, it is an epitheliotropic virus, which means that it has a preference for epithelium. Within the virus is a basic capsid that encloses a circular DNA genome. Three hundred and sixty copies of the main capsid chain, which is referred to as L1, are included in the virus. There is a second capsid chain, which is referred to as L2, that is located within the virus and may assist with the packaging of the DNA. L1 chains are responsible for the formation of 72 pentameric *capsomeres*, which subsequently engage in mutual interaction with one another utilizing long flexible tails [1-7].

Genome of HPV

The HPV genome consists of precisely 8,000 base pairs. They can remain infectious in damp environments for extended periods because they are relatively stable. There are three functional coding areas present in the virus. The long control region (LCR), also called the noncoding regulatory region (NCR), sits between the two genes that code for early and late viral functions, respectively [6].

The timing of a gene's functional activity determines whether it is considered late or early in the classification system. The HPV genome consists of two genes that code for late proteins (L1 and L2) and six genes that code for early proteins (E1, E2, and E4-E7). As the differentiated process progresses, E4, E5, E6, and E7 expression begin, whereas E1, E2, E5, and E7 expression persist. Finally, during the differentiation process, L1 and L2 are expressed. The early genes play a crucial role in cellular transformation, transcriptional regulation, and DNA replication, while the late genes are responsible for encoding capsid proteins. E1, E2, E6, and E7 are commonly expressed in cervical tumors caused by HPV.

In the presence of high-risk HPV E6/E7, cervical cancer cannot advance at a faster rate. These oncoproteins aid in tumor formation, contribute to genomic instability, and participate in other cancer-related activities. Once an HPV infection establishes its root, the viral genome stays in the epithelium's basal layer. Infected cells differentiate along the epithelium, a process that also triggers high-level replication of viruses and gene expression. At some point, the virion is ready to be released from the cell. The expression of viral genes is associated with the stages of epithelial development [8].

HPV replication and pathogenesis in relation to cervical cancer

Because HPV causes both benign and malignant lesions, ranging from those that are self-limiting to those that spread throughout the body, and because it affects so many people, understanding how HPV works has been challenging [9]. Oncogenic human viruses infect the basal epithelium; despite the high incidence of infection, most infections resolve spontaneously, while a small proportion of infected people tend to establish long-term persistent infections. While impaired immune function may exacerbate cervical cancer, the most significant risk factor is a chronic HPV infection, which can lead to the disease's growth and progression [5].

In biological evolution, HPV induces persistent infections by escaping host immunity and defense mechanisms through several processes. These include an absence of inflammation, lack of viral-induced necrosis or cytolysis, absence of a blood-borne or viremic phase, inadequate access to vascular and lymphatic channels, as well as lymph nodes where immune responses are initiated. Additionally, they possess mechanisms for inhibiting interferon synthesis and receptor signaling [6-10].

One step in the process of keratinocyte production is when HPV uses microscopic pores to access epithelial tissues. The keratinocytes in the spinous layer can replicate viral DNA and facilitate an action that enhances gene expression. Unique to keratinocytes, a type of squamous epithelial cell that lines the cervix, is the expression of viral genes [9,10].

For instance, HPV can attach itself to several components of the body's cellular lining, such as heparin sulfate proteoglycans (HSPGs) on the basement membrane or epithelial cell surface, or even to the extracellular matrix (ECM) via laminin-332. At the same time, complexes of HSPG/growth factor and HPV can activate epidermal growth factor receptors (EGFRs) and keratinocyte growth factor receptors (KGFRs). The phosphoinositide 3-kinase pathway is one possible intracellular signaling cascade that these receptors can activate [10].

The virion undergoes a conformational change just after it interacts with HSPGs. Next, the HPV establishes a link with the α6 integrin, which initiates an additional intracellular signaling cascade. After the virus undergoes conformational changes and signaling, it is transported within the cell through clathrin- or caveolae-mediated endocytosis. The virus enters the cell and travels to the nucleus, which contains the DNA molecule [11]. The process of viral replication results in the production of approximately 100 copies of HPV DNA outside of the chromosomes in each cell. This is achieved by stimulating viral gene expression and speeding up cell division [10,11].

Crucial at this point are the E1 and E2 proteins. Together, these two proteins recruit cellular polymerases and auxiliary proteins and regulate DNA replication through their complex formation. They accomplish this by joining together to produce a complex that attaches to the processes of viral replication [9-11].

A better understanding of the functions played by E4 and E5 in controlling the virus's late stages needs additional investigation. Proteins E6 and E7 aid in viral reproduction and dissemination. To construct its icosahedral capsid, the virus must first breach the suprabasal layers and initiate the production of its late viral genes. After all this work, infectious virions are ready to infect other cells once they are released [9,11].

Risk determinants

**Figure 1:**
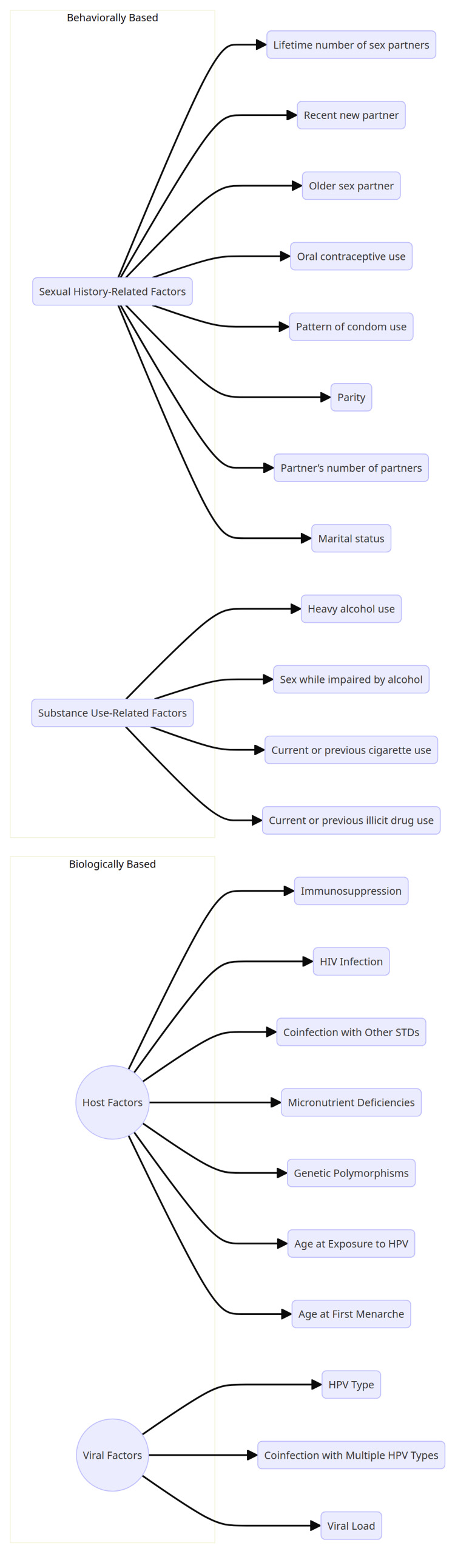
Risk factors known or postulated to be associated with HPV infection or HPV-related disease. Source: [12]. HPV, human papillomavirus

One helpful categorization of HPV risk factors, as shown in Figure 1, was based on biological or behavioral variables. Biological risk factors, which comprise both HPV virus features and intrinsic host variables that influence the immune response, mostly influence oncogenesis pathway downstream transitions rather than HPV acquisition risk [11,12].

Immunosuppression and other forms of immunodeficiency, as well as other sexually transmitted diseases (STDs) like Chlamydia trachomatis and herpes simplex virus, are host variables that influence the immune response. It is thought that the most critical aspects when it comes to biological risk factors of HPV are the type of virus and viral load. Furthermore, aging is considered an inherent host component that requires further investigation. More than three-quarters of all adolescents and young adults contract HPV between the ages of 15 and 25, according to a meta-analysis of research [12].

When developing risk-based HPV vaccination programs, it is crucial to take behavioral risk factors into account because they have a significant impact on the likelihood of infections. Some behavioral risk factors include insufficient genital cleanliness, a history of substance misuse, and specific details about one's sexual history, such as the number of partners, features of those partners, parity, and the use of contraception [12,13].

Route of transmission

Studies have shown that the acquisition of HPV infection takes place immediately after the beginning of sexual activity. On the other hand, a prospective study reveals that the recurrent incidence of infection is 40% within 24 months of the initial sexual encounter. It is estimated that the incubation time following an HPV infection can range anywhere from two weeks to eight months, with the majority of symptoms showing between two and three months after the infection. HPV can be transferred even when an infected person does not exhibit any signs or symptoms [14].

Transmission usually occurs with sexual intercourse but can also occur following non-penetrative sexual activity. Another route of nonsexual transmission of genital HPV is perinatal transmission at the time of birth from the infected mother, either by direct contact with the infected maternal genital tract or by ascending infection, especially after premature rupture of membranes. However, if the mother is found to be infected with HPV, restricting breastfeeding fails to demonstrate high-risk HPV-DNA transmission. One study shows that children cannot become infected with HPV via swimming pools, while another study by de Martino et al. shows HPV can be transmitted through infected towels or other objects. However, there is a conflicting opinion about fomite transmission [14].

Clinical pictures

Many HPV infections do not cause any noticeable symptoms at all. Period problems can manifest in a variety of ways, including lighter-than-usual bleeding or spots, heavier-than-usual menstrual bleeding, increased discharge from the vagina, agony during sexual activity, abnormal bleeding after the menopause, and constant pelvic and/or back pain that does not go away. On the other hand, they could be signs that HPV has progressed to cervical cancer [15].

The most crucial sign of HPV is the appearance of warts on the genitals, which can be flat lesions, little lumps that look like cauliflowers, or tiny stem-like protrusions. Cauliflowers are another appearance of warts. Genital warts can spread to the anogenital skin as well as the mucous membrane. Although few people report pain or discomfort from genital warts, others report itching and discomfort. However, warts on the vagina, cervix, or near the anus are other possible locations for genital growth. Among female genitalia, the vulva is where the vast majority of warts develop. Conversely, men with genital warts will observe the growths on their penis, scrotum, and the area around their anus [15].

Additional testing may be conducted after a cervical cancer diagnosis to ascertain metastasis or the spread of cancer to other areas of the body. The term for this is staging. To effectively treat cervical cancer, accurate tumor staging is required [16]:

- Stage 0: Carcinoma in situ in the innermost lining of the cervix - Stage I: Confined to the cervix - Stage II: Spread beyond the uterus but not to the lower third of the vagina or pelvic wall - Stage III: Spread to the lower third of the genital tract or pelvic wall, causing hydronephrosis or nonfunctioning kidney - Stage IV: Spread beyond the true pelvis or into the mucosa [17]

HPV diagnosis

The Pap test cytology was considered the gold standard for diagnosing and preventing cervical cancer in contemporary healthcare systems. Premalignant and malignant alterations, as well as viral diseases such as herpes and HPV infection, can be identified by the use of the Pap smear screening for cancer. If the test is positive, additional confirmatory testing, such as a colposcopy, a cervical biopsy, and DNA tests such as polymerase chain reaction (PCR), are required [18,19].

A diagnostic method for detecting viral DNA (the HPV DNA test) has been developed in response to the link between cervical cancer and the HPV. These approaches have the potential to be utilized as a primary means of screening for cervical cancer, and they can either replace or supplement the presence of the Pap test. The effectiveness of the HPV DNA test is superior to that of the Pap test in the detection of CIN2 or higher. The reason behind this is that unlike the Pap smear, which has a sensitivity ranging from 44% to 74% (with an average of 53%), the HPV DNA test achieves 100% sensitivity throughout the trial [19].

Women aged 25 years or above can undergo a single HPV DNA test, authorized by the Food and Drug Administration, as the primary screening method for assessing cervical cancer risk. Despite this, HPV testing can discover a large number of infections that do not cause cervical dysplasia or cancer. It is possible to achieve an exponential and reproducible increase in the number of HPV sequences that are present in biological materials with the use of a selective target amplification assay known as PCR [20].

 Both the Pap test, which examines cervical cells for abnormalities, and the HPV DNA test, which detects the presence of HPV DNA, are simultaneously conducted in a screening process known as co-testing. This approach is superior at identifying cervical abnormalities when compared to screening alone using the Pap test. However, it is associated with a higher rate of false positives as compared to strictly utilizing the Pap test. It may be necessary to perform tests more often and utilize more intrusive diagnostic methods if abnormal results are discovered [20]. Colposcopy is utilized for the examination of the cervix, the vagina, and in certain cases, the vulva. Furthermore, it can be utilized for the collection of biopsies from any suspected lesions that are indicative of neoplasia [20,21].

At first glance, the cervix appears to be normal. Additionally, the acetic acid test is administered to suspicious lesions to demonstrate the extent of subclinical genital HPV-associated lesions, identify lesions for target biopsy, and demarcate lesions during surgical therapy [21].

Epidemiology

Of all cancers affecting females, cervical cancer ranks fourth worldwide. Although Saudi Arabia does not have a nationwide screening program, cervical cancer is still quite uncommon among Saudi women, with a reported incidence of only 12% [1]. The World Health Organization (WHO) has calculated that 5,700,000 women die from cervical cancer every year, making it the seventh leading cause of cancer in women. According to WHO, cervical cancer is a real possibility for approximately 6.5 million Saudi Arabian women over the age of 15 years. In Saudi Arabia, malignancies caused by HPV are responsible for an estimated 55 female deaths each year [1,2].

During a cross-sectional study at a tertiary care hospital in Jeddah from October 2017 to April 2018, researchers utilized serological and vaginal swab techniques to establish a correlation between HPV infection and cervical cancer. Vaginal swabs from seven of the 199 females evaluated positive, as shown in Table 1.

**Table 1:** Prevalence of human papillomavirus in a convenience sample of females attending a gynecological clinic in Jeddah. Source: [1]

	Tested samples, *n* (%)	HPV positive, *n* (%)
Total number (%)	119	7 (5.9%)
Nationality		
Saudi, *n* (%)	100 (84.0%)	6 (6.0%)
Non-Saudi, *n* ​​​​​(%)	19 (16.0%)	1 (5.3%)

As shown in Table 2, there were 16 positive serum samples out of 966 collected from clean blood donors for the HPV seroprevalence [1]. 

**Table 2:** Seroprevalence of human papillomavirus in healthy blood donors (N = 966). Source: [1]

	Tested samples, *n* (%)	HPV-positive, *n* (%)
Total number (%)	966	16 (1.7%)
Nationality		
Saudi, *n* (%)	541 (56.0%)	9 (1.7%)
Non-Saudi, *n* (%)	425 (44.0%)	7 (1.7%)

HPV awareness in Saudi Arabia

Although cervical cancer is preventable and curable, the majority of women in developing countries, including Saudi Arabia, attend clinics with advanced stages that require extensive treatment, such as radiotherapy, chemotherapy, and surgery [22].

Delay in diagnosis may decrease the survival rate. Therefore, health education and awareness are crucial, particularly for educating women about the importance of screening and regular examinations, including Pap smear tests. Several investigations have demonstrated, however, that there is a dearth of education on HPV infection, screening, and vaccination among Saudi healthcare providers and the general Saudi public [22,23].

For instance, a descriptive cross-sectional study conducted in four main Riyadh healthcare facilities from January 2016 to June 2016 found that the Saudi population's level of knowledge and awareness regarding Pap smears as cervical cancer screening tests was unsatisfactory. The result of this study showed Pap smears were unfamiliar to 234 of the 507 women who took part in the study. The number of women who knew about it was 273 (53.9%). There was a sub-question asking about the sources of knowledge for women who answered yes. The results showed that 57.1% of women obtained their knowledge during hospital visits for obstetrics and gynecology reasons, while 15.4% acquired it through health facilities and staff. Additionally, 9.9% gained knowledge from media sources, 9.2% from friends, and 8.4% from posters or leaflets [23]. 

Another question asked in the survey was if they knew the importance of Pap smear. From the total of 507 women, 312 knew about the importance of Pap smear. Also, this study showed that only 24.9% of women had had the Pap smear before, while 75.1% did not. Finally, the women included in the survey were also asked if their physician advised them to undergo a Pap smear. The results were 75.5% for never, 15.6% for sometimes, and 8.9% for always [23].

HPV management

The crucial management for HPV infection is to treat health problems introduced by HPV infection, such as genital warts and cervical precancer. Management for cervical pre-cancerous includes routine Pap tests and follow-up as needed to identify any abnormal activity before the cancer develops [24].

A colposcopy, which uses a device to magnify the cervix and obtain biopsies for any regions that look suspicious, is recommended if an atypical HPV or Pap test is found. It is recommended to remove any precancerous lesions found during a colposcopy. Different procedures are used. Examples of these procedures include freezing (also known as cryosurgery); laser treatment; surgical removal; loop electrosurgical excision procedure (LEEP), which involves removing a thin layer of a segment of the cervix; and cold knife conization, which involves removing a cone-shaped piece of the cervix [24,25].

Immunology of natural HPV infections

Two mechanisms exist inside the immune system that can eradicate HPV infections. The first line of defense against virus entry into epithelial cells is the humoral reaction, which generates antibodies that neutralize the virus. It takes around six to 18 months for this immune response to develop, and serological rates are modest. However, around 70% of people do increase the detectable rate of anti-proteins that target a specific L1 epitope [25,26].

While these antibodies do an excellent job of protecting basal keratinocytes against first infections, they are not enough to stop secondary infections. After making touch with the basal membrane, the HPV penetrates the cell and binds to the basal membrane via an interaction with α6 integrin, a naturally occurring part of the hemidesmosome complex. Specifically, laminin 5 is bound to the L1 portion of the virus. Later on, the virus is taken in by the body after being transmitted to α6 integrin [26,27].

There is still uncertainty regarding the process of the internalization mechanism. The difficulties in reacting with type-specific anti-L1 antibodies can be explained by the fact that the epithelial cell peels the capsid after internalization, thereby losing L1 and L2. Cytotoxic T-cells are responsible for HPV cellular clearance; they interact with cells that are infected by recognizing expressed viral proteins (such as E6 and E7) [26].

HPV vaccines

The combination of HPV vaccination, which prevents infection, with regular cervical cancer screenings, which detect abnormalities early, synergistically reduces the incidence of the disease. The Advisory Commission on Immunization Practices (ACIP) recommends that the optimal age for HPV vaccination is 12 or 11 years of age, but it can be administered as early as nine years old. Adults can use the vaccine for up to 45 years for missed shots, and it is an essential tool in the fight against cervical cancer. Men can reduce the incidence of HPV-related external genital warts (EGWs) by 90.4% when they receive the vaccine [6,11,16,18]. Despite this, there is evidence that 85.6% of vaccinated men are protected from HPV infection [6,11,16,18]. By activating cellular immunity via dendritic cell (DC) and specific to antigen T-cell activity, therapeutic vaccination aims to remove subclinical HPV-associated disease. Infected cells lose their respective proteins when the HPV L1 and L2 genes are inserted into their genome. The HPV prophylactic vaccination produces antibodies that neutralize these contaminated cells, but they are unable to reach them. Because of this, the HPV vaccine will not protect against infections, abnormal cervical glands, or EGWs [28,29].

By introducing the L1 gene into a host and then stimulating the host to produce an excess of L1 proteins, recombinant technology enables the creation of HPV vaccines. Like viruses, the L1 proteins can self-assemble into very large particles (VLPs). Due to the absence of viral DNA, very low-density particles (VLPs) are neither infectious nor carcinogenic, despite their size and form being similar to that of the HPV virion [28,29].

According to Virus Recumbent, the HPV vaccination is classified into many types, which are used as prophylactics and treatments for HPV infection, including protein vaccines, live vector vaccines, nucleic acid vaccines, and DC-based vaccines [28]. In the context of live vector (viral-based vaccines), the vaccinia virus was used as a therapeutic vaccine by inducing lysis of infected host cells, which is highly immunogenic in stimulating T-cell responses. Eight patients with advanced cervical cancer were found to express E6 and E7 proteins in HPV 16 and 18 types. This was the first clinical trial to use live recombinant vaccinia virus as tissue antigen (TA)-HPV. The toxicity that patients experience after receiving a single dosage of TA-HPV is minimal and manageable. Moreover, cytotoxic T lymphocytes (CTLs) specific to HPV are produced in response to the TA-HPV vaccine in 28% of individuals. Two participants in the study did not develop cancers after 15 and 21 months of TA-HPV immunization, respectively. There are safety concerns that make viral vectors unsuitable for use in individuals with impaired immune systems [28].

DNA vaccines utilize plasmid DNA to introduce a particular gene into the host cell, where antibodies are produced by translation and transcription. It binds to major histocompatibility complex (MHC) classes I and II, which activate both cellular and humoral immune responses. Furthermore, to accomplish a very effective DNA vaccination, the circumstances discussed next must be met [28-30].

Vaccines administered intradermally or through the muscle layer should first include an adequate quantity of DNA to activate the immune cell. Second, there is enough supply of antigen-presenting cells (APCs) to absorb the antigen of interest. Third, antigen processing and presentation depend on effective APC. Finally, APC-mediated complete activation of T-cells. Repetition of the DNA vaccination does not result in the production of neutralizing antibodies.

Protein vaccines dominantly lead to more antibody production than cytotoxic T-cell protein vaccines. All possible epitopes (part of the antigen where the antibody attaches to it) can be extracted via processing by APCs. However, patients with cervical cancer have demonstrated effective immune responses to DC-based vaccinations that target HPV 16 and/or HPV 18 E7. In patients with cervical cancer, full-length E7-pulsed autologous DCs lysed HPV-infected cancer cells and generated E7-specific CD4+/CD8+ responses [30-33].

Gardasil and Cervarix are two of the most widely used vaccines today, offering protection against HPV types 6, 11, 16, and 18. Gardasil inhibits the growth of cancerous tumors in the vagina, vulva, and cervix as well as genital warts caused by types 11, 16, and 18, and the recurrence of HPV infection. However, as a bivalent vaccine, Cervarix provides defense from both HPV types 16 and 18. Vaccines containing virus-like particles (VLPs) made with recombinant DNA activate the immune system and generate antibody titers that are more potent than those caused by natural infection. Both vaccines contain the L1 protein part of the viral capsids [31].

Although vaccines are undeniably helpful in lowering the incidence of HPV infectious illnesses, there are differences in how the security of the HPV vaccine has been assessed. Among the most reported side effects of HPV vaccination include orthostatic intolerance, ongoing pain with paraesthesia, fatigue, headaches, and weariness, which can range from mild to severe. Although demyelinating diseases are less common following immunization, they can still occur. Also, the chance of having Guillain-Barré syndrome is less than one case per one million vaccinated, according to the most recent study by the Global Advisory Committee for the Safety of Vaccines (GACVS) [32-35].

Efficacy of vaccines

In recent years, the impact of the HPV vaccination in real-world scenarios has been more apparent, particularly for women living in countries with high vaccination rates who obtain immunizations before being exposed to HPV. Maximum reductions of around 90% have been reported for HPV type 6/11/16/18 infections, 90% for genital warts, 45% for low-grade cytological cervical abnormalities, and 85% for high-grade histologically confirmed cervical abnormalities. These findings have been reported previously. At least one dose of the HPV vaccine was associated with an efficacy rate of 83% to 96.1% [36-38].

Researchers in Denmark found a nonsignificant increase in the rate of cervical cancer in women who were vaccinated between the ages of 20 and 30 years, according to new statewide cohort research. On the other hand, there was an 86% decrease in the incidence of cervical cancer among 16-year-olds and younger and a 68% decrease among older teenagers. According to a study conducted in the United States [39], there was a statistically significant drop in the percentage of women who had been vaccinated against one or more of the four diverse types of vaccines.

The incidence of HPV infections decreased by 80.9% for nine-valent vaccine-type HPV infections and 68.8% for five-valent vaccine-type infections among women who had received at least one dose of the HPV vaccination. This was except for HPV type 6/11/16/18 infections, which were more prevalent in nine valent vaccine-type infections. However, the only percentage of unvaccinated women who had one or more of the four-valent vaccine-type HPV infections (a decline of 40.1%) and the five-valent vaccine-type HPV infections (a decline of 5.6%) was noteworthy. The only exception to this was the HPV type 6/11/16/18 infection, which had a decline of 57.6% among the nine-valent vaccine-type HPV infections. The most recent research from India reported a 95.4% success rate of the vaccination against HPV types 16 and 18 persistent infections in a single-dose cohort. The 95% confidence interval for this result was from 85.0% to 99.9%, respectively. The effectiveness of Gardasil 9 against HPV-type-associated cervical malignancies is 88% in Asia, 87% in Australia, 91% in Europe, 90% in Latin America and the Caribbean, and 92% in Africa and North America, according to the findings of researchers [37-39].

Awareness of HPV vaccines among Saudi women

A study by Alhusayn et al. found that 70.6% of Saudi parents of patients at Family Medicine Paediatric Clinics at King Faisal Specialist Hospital and Research Centre in Riyadh were unaware of the HPV vaccine. The majority of participants did not associate HPV with cervical cancer, with 38.8% and 37.8% not knowing or not associating it with cervical cancer. Only 28.6% were aware of the vaccine's potential to prevent cervical cancer, and 89.5% did not receive it for themselves or their children [40,41].

WHO updates recommendations on HPV vaccination schedule

The optimization of the HPV vaccination schedule is expected to enhance vaccine accessibility. This will provide nations with the opportunity to increase the number of girls who can receive the vaccination and reduce the strain of the often challenging and costly follow-up required to complete the vaccination series. Strengthening HPV vaccination programs, putting them into action fast, and halting coverage reductions are all necessary for countries. The current WHO recommendation for girls aged 9 to 14 is to receive two doses of the vaccine, administered at ages 9 and 14. Girls and women aged 15 to 20 years can receive one or two doses, while women over 21 should get two doses spaced six months apart. The policy paper underscores the critical importance of promptly vaccinating HIV-positive individuals. Individuals with weakened immune systems should receive two vaccine doses, and a third dose may be considered based on their immune response and medical condition. When it comes to immunizations, the primary population of interest is girls between the ages of nine and 14 years, before they start having sexual relations. It is recommended that secondary targets, such as older girls and boys, be vaccinated whenever it is feasible and cost-effective [40].

## Conclusions

HPV has been identified as an essential etiology of cervical cancer. HPV infection can be transmitted asymptomatically, meaning that individuals can spread the virus without showing any signs of being infected. Raising public awareness of the risk factors associated with HPV infection is crucial for early detection, prevention, and ultimately reducing the burden of cervical cancer. This study emphasizes the crucial role of cervical cancer screening and the preventive advantages of HPV vaccination. Regular screening for cervical cancer, such as Pap smears and HPV testing, can help detect precancerous lesions early on. Moreover, vaccination against HPV has been shown to significantly reduce the risk of developing cervical cancer.
